# Foot ulcer simulation training (FUST): are podiatrists FUST with long-term clinical confidence?

**DOI:** 10.1186/1757-1146-6-S1-O22

**Published:** 2013-05-31

**Authors:** Peter A Lazzarini, Vanessa Ng, Patricia M Régo, Suzanne S Kuys, Scott Jen

**Affiliations:** 1 Allied Health Research Collaborative, Metro North Hospital & Health Service, Queensland Health, Brisbane, Queensland, 4032, Australia; 2 Department of Podiatry, Metro North Hospital & Health Service, Queensland Health, Brisbane, Queensland, 4032, Australia; 3 School of Clinical Sciences, Queensland University of Technology, Brisbane, Queensland, 4059, Australia; 4 School of Medicine, The University of Queensland, Brisbane, Queensland, 4072, Australia; 5 Clinical Skills Development Service, Centre for Healthcare Improvement, Queensland Health, Brisbane, Queensland, 4029, Australia; 6 Musculoskeletal Research Program, Griffith Health Institute, Griffith University, Gold Coast, Queensland, 4222, Australia; 7 Department of Podiatry, West Moreton Hospital & Health Service, Queensland Health, Ipswich, Queensland, 4305, Australia

## Background

Foot ulcers are a leading cause of diabetes-related hospitalisations. Clinical training has been shown to be beneficial in foot ulcer management. Recently, improved self-confidence in podiatrists was reported immediately after foot ulcer simulation training (FUST) pilot programs. This study aimed to investigate the longer-term impacts of the FUST program on podiatrists’ self-confidence over 12 months in a larger sample.

## Methods

Participants were podiatrists attending a two-day FUST course comprising web-based interactive learning, low-fidelity part-tasks and high-fidelity full clinical scenarios. Primary outcome measures included participants’ self-confidence measured pre-, (immediately) post-, 6-month post- and 12-month post-course via a purpose designed 21-item survey using a five-point Likert scale (1=Very limited, 5=Highly confident). Participants’ perceptions of knowledge gained, satisfaction, relevance and fidelity were also investigated. ANOVA and post hoc tests were used to test any differences between groups.

## Results

Thirty-four participants completed FUST. Survey response rates were 100% (pre), 82% (post), 74% (6-month post), and 47% (12-month post). Overall mean scores were 3.13 (pre), 4.49 (post), 4.35 (6-month post) and 4.30 (12-month post) (*p* < 0.05); post hoc tests indicated no differences between the immediately, 6-month and 12-month post group scores (*p* > 0.05). Satisfaction, knowledge, relevance and fidelity were all rated highly.

## Conclusion

This study suggests that significant short-term improvements in self-confidence to manage foot ulcers via simulation training are retained over the longer term. It is likely that improved self-confidence leads to improved foot ulcer clinical practice and outcomes; although this requires further research.

